# Partial rewarding during clicker training does not improve naïve dogs’ learning speed and induces a pessimistic-like affective state

**DOI:** 10.1007/s10071-020-01425-9

**Published:** 2020-09-08

**Authors:** Giulia Cimarelli, Julia Schoesswender, Roberta Vitiello, Ludwig Huber, Zsófia Virányi

**Affiliations:** 1 Clever Dog Lab, Comparative Cognition, Messerli Research Institute, University of Veterinary Medicine Vienna, Medical University of Vienna, University of Vienna, Veterinaerplatz 1, Vienna, 1210 Austria; 2 Domestication Lab, Konrad Lorenz Institute of Ethology, University of Veterinary Medicine Vienna, Veterinaerplatz 1, 1210 Vienna, Austria; 3 R(D)SVS, University of Edinburgh, Easter Bush, Midlothian, EH25 9RG Scotland; 4 Scotland’s Rural University College (SRUC), Easter Bush, Midlothian, EH25 9RG Scotland; 5 UMR PRC, INRA, CNRS, IFCE, Université de Tours, 37380 Nouzilly, France

**Keywords:** Clicker training, Partial rewarding, Domestic dog, Operant conditioning, Cognitive bias, Personality

## Abstract

Clicker training is considered a welfare-friendly way of teaching novel behaviors to animals because it is mostly based on the positive reinforcement. However, trainers largely vary in their way of applying this training technique. According to the most, a reward (e.g., food) should follow every click, while others claim that dogs learn faster when the reward is sometimes omitted. One argument against the use of partial rewarding is that it induces frustration in the animal, raising concerns over its welfare consequences. Here, we investigated the effect of partial rewarding not only on training efficacy (learning speed), but also on dogs’ affective state. We clicker-trained two groups of dogs: one group received food after every click while the other group received food only 60% of the time. Considering previous evidence of the influencing role of personality on reactions to frustrated expectations, we included measurements of dogs’ emotional reactivity. We compared the number of trials needed to reach a learning criterion and their pessimistic bias in a cognitive bias test. No difference between the two groups emerged in terms of learning speed; however, dogs that were partially rewarded during clicker training showed a more pessimistic bias than dogs that were continuously rewarded. Generally, emotional reactivity was positively associated with a more pessimistic bias. Partial rewarding does not improve training efficacy, but it is associated with a negatively valenced affective state, bringing support to the hypothesis that partial rewarding might negatively affect dogs’ welfare.

**Electronic supplementary material:**

The online version of this article (10.1007/s10071-020-01425-9) contains supplementary material, which is available to authorized users.

## Introduction

Clicker training is a widely used technique to teach novel behaviors to dogs and other species by combining two forms of learning: classical and operant conditioning (Ferster and Skinner 1957; Skinner 1969). During such training, the individual’s behavior is reinforced by associating a specific response to an arbitrary stimulus through a positive reinforcer as in operant conditioning, through the use of a sound (a click, conditioned stimulus and secondary reinforcer). The sound anticipates the reward delivery (unconditioned stimulus and primary reinforcer) as in classical conditioning. The reasons for using a clicker are various: it allows to fill the temporal delay between the response and the reward delivery (Pryor 1999; Feng et al. 2018), to work at a distance (Pryor 1999; Feng et al. 2018), and it has the advantage of being highly detectable (Chiandetti et al. 2016). The basic process of clicker training is simple: the animal shows the desired behavior, the trainer clicks, and then she/he delivers a reward.

The pairing between the secondary and the primary reinforcer can follow different schedules: in a continuous rewarding schedule, whenever the trainer clicks, the reward follows; whereas in a partial rewarding schedule, sometimes the secondary reinforcer is provided without being followed by the primary one (that is, the trainer clicks, but only sometimes the reward is provided). Most practitioners advocate the use of continuous rewarding, arguing that removing the primary reinforcer (e.g., food) would weaken the association with the secondary reinforcer that would become less effective in marking the correct behavior and in signaling the arrival of the reward (Pryor 1999; Fernandez 2001; Clayton 2005). This last statement is based on the observation that upon a secondary reinforcer ceasing to be paired with a primary reinforcer, the association between the two gets extinguished (Zimmerman 1957, 1971; Egger and Miller 1962; Berger et al. 1965). However, considering that in a partial rewarding schedule the secondary reinforcer is again occasionally paired with a primary reinforcer, this extinction does not happen (D’Amato et al. 1958; Fox and King 1961; Armus et al. 1962). Instead, the learning curve of animals being reinforced continuously or only partially does not differ (D’Amato et al. 1958; Fox and King 1961; Armus et al. 1962).

In contrast, other practitioners promote the idea that partially omitting the food after each click can help to increase the individual’s motivation and attention, and ultimately, to improve training efficacy (McConnell 2014; Cecil 2016; but see Martin and Friedman 2011). Based on this, an individual whose correct behavior is marked by the secondary reinforcer but not always rewarded would learn a novel behavior more quickly than one who is always rewarded. The reasoning behind this is that a subject’s motivation and attention are higher if the secondary reinforcer is not always paired with the primary reinforcer, due to a potential activation of the “seeking” system (Wise 2004; Berridge et al. 2009). In particular, the anticipation of a reward (in this case the click) would activate the “seeking” system, which is supposed to mediate a higher motivation than the reward itself (mostly activating the “liking” system) (Berridge et al. 2009; Panksepp 2011). Following this reasoning, in a partial rewarding schedule, when the click is not followed by a reward, the “seeking” system would be activated more strongly than the “liking” system, resulting in an even stronger effect on attention and motivation than the click–reward pairing.

In addition, even though a technique might be effective, it could still have an emotionally negative impact on the subject. A clear example for this is the use of positive punishment: it is effective (an animal reduces the likelihood of showing a specific behavior), but the impact on an animal’s affective state can be detrimental (Schilder and van der Borg 2004; Blackwell and Casey 2006). Generally, training which is based mostly on positive reinforcement (as in the case of clicker training) has been associated with improved animal welfare (e.g., Gillis et al. 2012; Prescott and Buchanan-Smith 2003). However, small methodological differences within the use of positive reinforcement-based methods could have a diverse impact on an animal’s affective state (with a potentially smaller amplitude in comparison to positive punishment-based methods). For instance, the absence of a reward after a click might lead to the animal’s frustration, raising concerns over a potential negative impact on the animal’s affective state (Pryor 1999; Fernandez 2001; Clayton 2005). This is supported by former studies on operant conditioning showing that when the expectation of receiving a reward has not been met, rodents show behaviors suggesting a negative affective state (Cuenya et al. 2012; Burokas et al. 2012). Still, whether different ways of delivering a reward during clicker training would lead to different affective states has been, to date, overlooked.

Importantly, in humans and rodents, individual differences play a major role in determining the response to the omission of an expected reward (Carver and White 1994; Gross et al. 1998; Corr 2002; Cuenya et al. 2012). As such, emotional reactivity and its two components: sensitivity to rewarding (positive activation) and sensitivity to aversive experiences (negative activation, Carver and White 1994; Gray 1991) may affect this response. On the one hand, the omission of an expected reward should have a stronger impact on individuals with a higher score on negative activation, as they may perceive the absence of reward as a stronger punishment (see Gray’s Reinforcement Sensitivity Theory, e.g. Gray 1991). On the other hand, however, individuals scoring high on positive activation may be more sensitive to the reward per se, and therefore respond stronger to reward omission than animals with low positive activation (Corr 2002). To date, no study has specifically investigated the relation between reward omission and these two traits in dogs, even though it could have important practical implications, allowing the design of training methods tailored to the individual’s personality.

Despite the widespread use of the clicker to train dogs, experimental evidence regarding the use of partial rewarding is still lacking. To the best of our knowledge there was only one previous study, an unpublished Master thesis (Wennmacher 2007), using a within-subject design, in which two dogs were rewarded either 100% or 50% of the trials (while the click was always provided), in alternating sessions. In contrast to the aforementioned hypothesis of partial rewarding increasing motivation, the results showed that the frequency and the accuracy of the target behaviors were lower in those sessions in which food was provided only partially. In addition, Wennmacher (2007) reported the emergence of unwanted behaviors in the partial rewarding sessions, including avoidance and stress-related behaviors, potentially confirming a negative impact on dogs’ affective state. However, the very limited sample size and the within-subject design (that did not allow to measure potential carryover effects between one session and the next) renders the conclusions of this Master thesis premature and thus call for another experimental attempt.

In the present study, we aimed at investigating the effect of partial rewarding during clicker-based training sessions on training efficiency and dogs’ affective state. We compared two groups of pet dogs clicker trained to perform a novel task with 100% or 60% rewarding: one group was rewarded after each click while the other received a reward only after 60% of the clicks. We measured how many trials were needed to learn the novel behavior. Moreover, to quantify the affective state following training, we used a task measuring subjects’ reaction to an ambiguous situation (called “cognitive bias test”, Mendl et al. 2009). Previous research on a variety of species has indicated that this procedure is a reliable indicator of the valence (positivity or negativity) of an animal’s affective state (Harding et al. 2004; Mendl et al. 2009; Burman et al. 2011). In addition, since affective responses to reward omission seem to be influenced by personality-related factors like emotional reactivity (Gross et al. 1998; Corr 2002; Cuenya et al. 2012), we provided owners with a questionnaire developed and validated by Sheppard and Mills (2002) to measure such personality traits, specifically in dogs. Using the components extracted from the questionnaire, we assessed whether dogs’ emotional reactivity interacted with the treatment received and consequentially influenced the dogs’ reaction to the ambiguous stimulus.

Based on the argument that a partial rewarding schedule would increase motivation and attention by activating the “seeking” system (Wise 2004; Berridge et al. 2009), we hypothesized that partial rewarding during clicker training results in faster learning. However, considering the previous evidence showing no difference in terms of behavior acquisition between animals continuously or partially rewarded (D’Amato et al. 1958; Fox and King 1961; Armus et al. 1962), we formulated an alternative hypothesis according to which dogs would learn a novel behavior (i.e., two paws on a wooden board) at a comparable speed, independently from the rewarding schedule (i.e., the null hypothesis). In addition, based on previous evidence suggesting that reward omission promotes frustration (Gross et al. 1998; Corr 2002; Cuenya et al. 2012), we hypothesized that a partial rewarding schedule results in a short-term more negative judgement of an ambiguous stimulus (that is, a more negative affective state), than continuous rewarding. Moreover, we predicted that dogs with a more reactive personality [either scoring high in negative activation, as according to Gray (1991), or in positive activation as according to Corr (2002)] will be more negatively influenced by the absence of reward after a click; therefore, showing a more pessimistic response in the cognitive bias test, than less reactive dogs.

## Materials and methods

### Subjects

Thirty pet dogs were included in the present study and were assigned to two groups, counterbalanced for sex and age. No dog had previous experience with clicker training nor had they been trained to perform the target behavior. Fifteen dogs (9 males, 6 females, age mean ± SD = 42.33 ± 22.99 months, 1 Border Collie, 1 Australian Shepherd, 1 Border Terrier, 1 Hovawart, 1 Podenco, 1 German Shepherd, 9 mongrels) were assigned to a “100% Rewarding” group (see below), whereas 15 dogs were assigned to a “60% Rewarding” group (8 males, 7 females, age: mean ± SD = 44.07 ± 22.32 months, 1 Border Collie, 1 Gordon Setter, 1 Golden Retriever, 1 Labrador Retriever, 1 Chinese Crested dog, 10 mongrels). We used a mix of breeds (using a comparable number of working and companion breeds across the two groups) and mongrels to represent the average performance of a pet dog. In the case of one dog (belonging to the 60% group), due to problems with video recording, the Clicker test could not be coded, therefore he has only been included in the analyses of the Cognitive Bias test.

### Ethical statement

The methods applied do not qualify as animal experimentation according to Austrian laws (Animal Experimentation Law 2012). The experimental procedures were approved by the institutional ethics and animal welfare committee of the University of Veterinary Medicine Vienna in accordance with GSP guidelines and national legislation (approval number: ETK-06/03/2017).

### Overall experimental design

Dogs naïve to clicker training were recruited through the Clever Dog Lab (University of Veterinary Medicine, Vienna) database. The study consisted of three consecutive stages, performed on different days (see Table 1). In Stage 1, dogs were prepared for the “Cognitive Bias test” by being trained to discriminate between two different locations: one positive, where food was always present, and one negative, always empty (training phase). In Stage 2, dogs were included in a training procedure (“Clicker training”, divided into an association phase + training phase) where they were taught to put two paws on a target (i.e., wooden board) for at least two seconds. Once the subjects had reached the criterion of reliably putting the two front paws on the target, they were invited to a final stage (Stage 3) consisting of a Cognitive Bias refreshment (to be sure they still reliably discriminated between the positive and negative location), followed by a Clicker training (testing phase, one session of max. 40 clicks or max. 10 min), followed by the testing phase of the “Cognitive Bias test” (test duration: 45 min). Breaks of 10 min were divided between the three tests conducted on the last day, specifically, the testing phase of the “Cognitive Bias test” started 10 min after the last Clicker training session. Stage 3 did not last more than 90 min in total. The whole experiment took three to six testing days, with approximately a week between appointments (see Table 1 for information on how many days were needed for each stage). We used the Clicker training phase (Stage 2) to measure the learning speed. We decided to test their affective state only once they had all reached the final criterion in order to have them all at the same training level. Because we could not predict when the dog would have reached the final criterion, and since the Cognitive Bias refreshment phase needed to be conducted before the Cognitive Bias test, we could not conduct the Cognitive Bias test immediately after a last Clicker training session. If we were conducting the Cognitive Bias refreshment phase and testing phase, one after the other, immediately after the last Clicker training session, we would have had a large temporal delay between the treatment (that is, the Clicker training) and the Cognitive Bias testing phase, risking the weakening of a possible effect.

**Table 1:** Timeline

Stage 1 (1–2 days)	Stage 2 (1–3 days)	Stage 3 (1 day)
Cognitive Bias (training phase)	Clicker training (association phase and training phase)	Cognitive Bias (refreshment phase) + Clicker training (testing phase) + Cognitive Bias (testing phase)

### Detailed procedures

#### Clicker training

##### Association phase

The aim of this phase was to establish an association between the primary and secondary reinforcer. The Clicker training was conducted in an experimental room (size: 3 × 4 m) with three cameras attached to the ceiling to have a full overview. Upon arrival, the experimenter (E) greeted the dog by talking gently to the dog and petting the dog if the dog was comfortable with it. The dog was free to move during the whole experiment. The dog could first explore the experimental room for five minutes before the onset of the experiment. After that, E demonstrated to the dog that she had food and rewarded the dog by throwing one treat to the dog whenever the dog approached E or by asking some simple commands the dog already knew (as previously told by the owner, O). This procedure was repeated five times. Then, E started to use the clicker and continued to ask for simple behaviors the dog had been trained on (e.g., sit, lay down), clicked when the dog performed the correct behavior, and immediately rewarded the dog afterward. The procedure was repeated five times (every correct behavior was followed by one click, followed by one reward). Previous studies have shown that five clicks, each followed by a treat, are enough to establish an association between the primary and secondary reinforcer (e.g. Chiandetti et al. 2016).

##### Training phase

The aim of this phase was to train the target behavior. Immediately after the association phase, E sat on the floor on one side of the room, opposite to the entrance, while O sat on a chair 1.5 m away from the door. The O was asked to fill out a questionnaire (see below) and ignore the dog. E kept food (i.e., small pieces of sausages) in a pouch placed on the side of her hip, then looked at the target and waited for the dog to show any of the following behaviors: (1) Looking at the target; (2) Moving one paw towards the target; (3) Making one step towards the target; (4) Sniffing the target; (5) Putting one paw on the target; (6) Putting both front paws on the target. E clicked in response to any of the aforementioned behaviors that were initially shown. In the following trial, E clicked for the same exhibited behavior unless the dog advanced within the progressive sequence of behaviors. For instance, E initially clicked when the dog sniffed at the target, and kept doing so in the following trials. However, if at any time the dog advanced in the sequence of behaviors by putting one paw on the target, then E clicked this novel behavior. This procedure was applied equally to all dogs of both groups.

Two sessions (maximum 10 min each or maximum 40 trials/clicks) per day were carried out with a break of five minutes between them. As previously mentioned, dogs were divided in two groups: “100% Rewarding group” (every click was followed by a reward) and “60% Rewarding group” (only 60% of the total number of clicks are followed by a reward). Rewarding in the 60% group adhered to a semi-randomized order, with a maximum of three consecutive rewarded clicks in a row, and never two consecutive nonrewarded clicks. In order to control for the amount of reward given to the subjects in the two groups, food was weighed before the beginning of a session and equally matched between the two groups (one sausage weighing 60 g was used per each dog per session). As the dogs in the 100% group could receive a higher number of pieces of food (more trials followed by a reward), these dogs received smaller pieces of food (on average 1.5 g/piece) than the dogs in the 60% group (on average 2 g/piece). For dogs in both groups, to ensure that all dogs received the same amount of food, independently from the number of clicks needed to reach the end of the session or from the group, leftover food was thrown on the floor after the last click, at the end of the session. A dog reached the training criterion to pass to the following phase once he/she had put both front paws on the target for five trials in a row.

Since the E was aware of subjects’ assignment to each group, at least we kept E blind to successive trials’ reward schedule: E was provided with a random sequence of 40 trials in each session, printed on paper and attached to her leg with tape (each row reported whether the trial was rewarded or not). E was sitting on the floor with crossed legs; the position of the list on E’s leg maximized visual access to it, consequently minimizing the temporal delay between the click and reward presentation. An additional blank paper with a small hole covered the list, save for one row (= 1 trial). During the clicker session, E would click, move the blank paper to reveal whether it was a rewarded trial or not, and reward/nonreward the trial accordingly. By using this procedure, we avoided that E would know in advance whether the next click would be a rewarded one or not.

Considering that dogs were naïve to such training procedures and unfamiliar to E, to avoid that the training situation was too artificial (see Feng et al. 2016), E did not refrain from communicating with the dog and behaved as spontaneously as possible (e.g., by sometimes praising the dog for having shown a correct behavior). However, considering that E could have shown such behaviors differently to dogs belonging to the different groups, we coded E’s behaviors and we controlled for them in the analysis (see below).

##### Testing phase

This phase took place on the last testing day (see Table 1). The procedure was the same as in the training phase. However, since dogs could already put both front paws on the target reliably, E started to reward behaviors that would eventually make the dog lay on the target (i.e., lowering the body posture, bowing with both front paws on the target). The session was terminated after 10 min or 40 trials, independently from having reached the final behavior.

#### Cognitive Bias test

This test consisted of a nonsocial spatial discrimination task adapted from Mendl et al. (2010) and Müller et al. (2012), and it was divided into a Training phase and a Testing phase.

##### Training phase

In the Training phase dogs learned to discriminate between two positions, one was always baited (P: Positive) while the other one was not (N: Negative). In each trial, E positioned a bowl either baited or nonbaited at one of two possible positions (P and N) equidistant from the dog. During bowl positioning, the O covered the eyes of the dog with her/his hands to avoid that the dog could watch the experimenter place the bowl, as this could influence the dogs’ bias. After placing the bowl, E went behind a visual barrier and signaled O to uncover the dog’s eyes and release the dog. Each dog received a maximum of 60 trials per day (sessions of 20 trials with a short break between them) including an equal number of positive and negative trials. The location of the baited bowl (left or right) was counterbalanced across subjects. The Training phase ended when the individual reached the criterion of being two seconds faster in reaching the bowl in the last three positive trials than in the last three negative trials.

##### Refreshment phase

The Refreshment phase was conducted on the last day of the experimental procedure, immediately before the last Clicker training. In this phase, dogs had a short training repetition of 20 training trials to see whether dogs could still discriminate between the positive and negative location.

##### Testing phase

The Testing phase was conducted after the last Clicker training. The Testing phase trials looked similar to those of the Training phase, with the difference that the bowl could be positioned in three additional unrewarded positions (probe trials), located between the positive and negative locations: near negative (NN), near positive (NP) and middle (ME). The Testing phase comprised 6 probe trials (two of each of the three kinds) interspersed within 20 standard trials (e.g., PPNN × NPN × NNP × PPNP × NPN × PNP × , where the x corresponds to a probe trial; for details, see Mendl et al. 2010 and Müller et al. 2012).

##### Trial procedure

O held the dog on the leash in front of her/him, while E sat behind a curtain, out of view of the subject. At the beginning of a trial, E brought the baited/nonbaited bowl to a specific location (P, NP, ME, NN, or N) while the subject had the eyes covered by O, went back behind the curtain and knocked on the floor to signal to O the possibility of releasing the dog. Once the dog was released and once it reached the bowl and eventually ate the food, it was called back by O to the starting position. O was blindfolded during the trials, but could remove the blindfold between trials.

### Video coding

To measure learning speed, we coded the number of trials until the dog learned the final behavior (i.e., putting both paws on the target) during the Clicker training. Moreover, considering the impossibility of having a double-blind experimental design, we also analyzed E’s behavior to control for its possible effect on differences between groups. More specifically, we analyzed the number of positive social behaviors shown by the trainer (i.e., praising, smiling, petting the dog; labeled as “E Affiliation”), the number of social negative behaviors (i.e., gently pushing the dog away with one hand from herself or the food pouch, saying “no” or “nein”, turning the head away from the dog; labeled as “E Rejection”), and the number of attention-gatherer cues (calling the name of the dog, snapping the fingers, clapping the hands and banging on the floor; labeled as “E Reactivation”).

As a proxy of pessimistic bias, we coded the latency of approaching the bowl: i.e., the time elapsed between the release from the lead and the dog putting its nose within 30 cm of the bowl (distance from which the dog could see if the bowl contained the reward or not) during the Cognitive Bias test (see below for details on how this variable was then used).

Videos from the Clicker training and the Cognitive Bias test were coded by different observers. Twenty-four percent of the videos from the Clicker training (in total 10 dogs) were independently coded by a second person. Inter-rater reliability between the main coder and the second one was good (“E Affiliation”: Intra-Class Correlation Coefficient (ICC) = 0.66, *F* (9, 4.93) = 7.61, *p* < 0.05; “E Reactivation”: ICC = 0.63, *F* (9, 9.83) = 4.69, *p* < 0.05) or excellent (“E Rejection”: ICC = 0.93, *F* (9, 9.11) = 25.4, *p* < 0.001) depending on the variable. Moreover, videos of the Cognitive Bias test were coded by an observer blind to group allocation. Sixteen percent of the videos from the Cognitive Bias test sessions (in total 260 session) were independently coded by a second person who was also blind to group allocation. Inter-rater reliability between the main coder and the second one was excellent (“Latency to reach the bowl”: ICC = 0.83, *F* (256, 253) = 11.2, *p* < 0.001). Videos were either scored by entering data on an excel sheet (Clicker training) or coded using Somolon Coder (@ András Péter) (Cognitive Bias test).

### Questionnaire

Owners were asked to fill an emotional predisposition questionnaire (developed and validated by Sheppard and Mills 2002) to assess whether the emotional reactivity of the dog might interact with the treatment received to influence dogs’ reaction to the ambiguous stimulus. The questionnaire consisted of 21 items which responses were recorded on a 5-point Likert scale ranging from “strongly disagree” (score 1) to “strongly agree” (score 5); see Supplementary Material for a complete list of the questions.

### Statistical analysis

Following Sheppard and Mills (2002), we conducted a Principal Component Analysis (PCA) with Varimax rotation on the 21 questionnaire responses provided by the O. The two extracted components (“Positive Activation” and “Negative Activation”, see below) were then used as predictors in the models (see below).

To analyze whether treatment (potentially mediated by emotional reactivity) influenced learning speed, we built a linear model with the number of trials to reach criterion as response variable (log-transformed) and the interactions between Group (100% vs. 60%), and the two emotional reactivity components as predictors. To control for E’s potentially biasing behaviors, we included “E Affiliation”, “E Rejection”, and “E Reactivation” as predictors (log-transformed). We originally included sex and age as predictors, but the model complexity resulted too high (calculated based on the number of estimated terms in relation to sample size). Considering that these two variables were not part of any hypotheses (and both and age and sex were counterbalanced across groups), to achieve an acceptable level of model complexity, we removed these two variables from the final model. We used an *F*-test to determine whether the full model differed from a null model only containing the three E behavioral variables (Forstmeier and Schielzeth 2011).

Latencies to reach the bowl coded during the Cognitive Bias test were used to calculate latency scores for each probe location as done in previous studies (Mendl et al. 2010; Müller et al. 2012), using the following formula:

$$\frac{\left({\text{latency to probe location}}-{\text{mean latency to positive location}}\right)*100}{({\text{mean latency to negative location}}-{\text{mean latency to positive location}})}.$$

The mean latencies to positive and negative locations were taken from the standard trials conducted during the test sessions. Such calculation was conducted to account for individual differences in running speed. We ran this calculation for each probe location (NP, ME, NN).

To analyze whether treatment influenced the performance in the cognitive bias test, we built a linear mixed model with the latency score as response variable. Group (100% vs. 60%) and Location (NP, ME, NN) were included as fixed effects, as well as the interaction between the two, to test whether a treatment effect was restricted to only some of the probe locations. Moreover, we included the interactions between the two questionnaire components and Group to test whether treatment effect was conditional to the dogs’ emotional reactivity. To control for E’s potentially biased behaviors, we included “E Affiliation”, “E Rejection”, and “E Reactivation” as predictors (log-transformed). Moreover, we controlled for subject’s sex and age. The identity of the subject was included as random effect.

Considering that the treatment could have influenced the dogs’ general speed to reach the bowl, we also ran a model using the raw mean latency to reach all locations as response variable. We used the same predictors and random structure as for the model analyzing the effect on the latency score. Moreover, to exclude that a possible difference between the two groups was due to other factors than the treatment itself, we ran a linear mixed model with the mean latency to reach the positive and the negative location during the refreshment phase of the Cognitive Bias test (conducted before the last clicker training session). The interaction between Group and Location, as well as the two questionnaire components, the three variables on E’s behavior (log-transformed), sex, and age were included as predictors. The identity of the subject was included as random effect. For all models conducted on the behavioral variables coded during the Cognitive Bias test, we used a likelihood ratio test to determine whether the full model differed from a null one only containing the three E behavioral variables as well as dog’s age and sex.

For all models, we *z*-transformed all continuous predictors (i.e., age, “E Affiliation”, “E Rejection”, “E Reactivation”, “Positive Activation”, “Negative Activation”) to a mean of zero and a standard deviation of one to obtain more easily interpretable model estimates (Schielzeth 2010). Models’ assumptions were checked by plotting residuals vs. fitted values (homogeneity of variance) and by means of qqplots of the models’ residuals (normality). Model stability was determined by removing individual cases one at a time (model 1) or individual dogs (models 2, 3 and 4) and comparing model estimates obtained from each subset to those obtained for the full dataset. Confidence intervals were determined based on parametric bootstrapping (*n* = 1000 bootstraps) based on random sampling (with replacement) of the individual subjects, allowing to define whether potential negative results could be due to the small sample size.

The PCA was conducted using SPSS v. 25 (IBM) while the models were fitted in R 3.6.2 (R Development Core Team 2019) using the function *lmer* of the package lme4 (version 1.1–21, Bates et al. 2015).

## Results

### Questionnaire

The PCA revealed two components (“Positive Activation” and “Negative Activation) that reflected the original components identified by Sheppard and Mills (2002). The two components explained 21.63% and 17.13% of the total variance (similarly to what was previously reported in the original publication) and were not correlated with one another (Pearson’s *R* = 0.00, *p* = 1.0); see Table S1 for components loadings (Supplementary Material).

### Learning speed

**Fig. 1:**
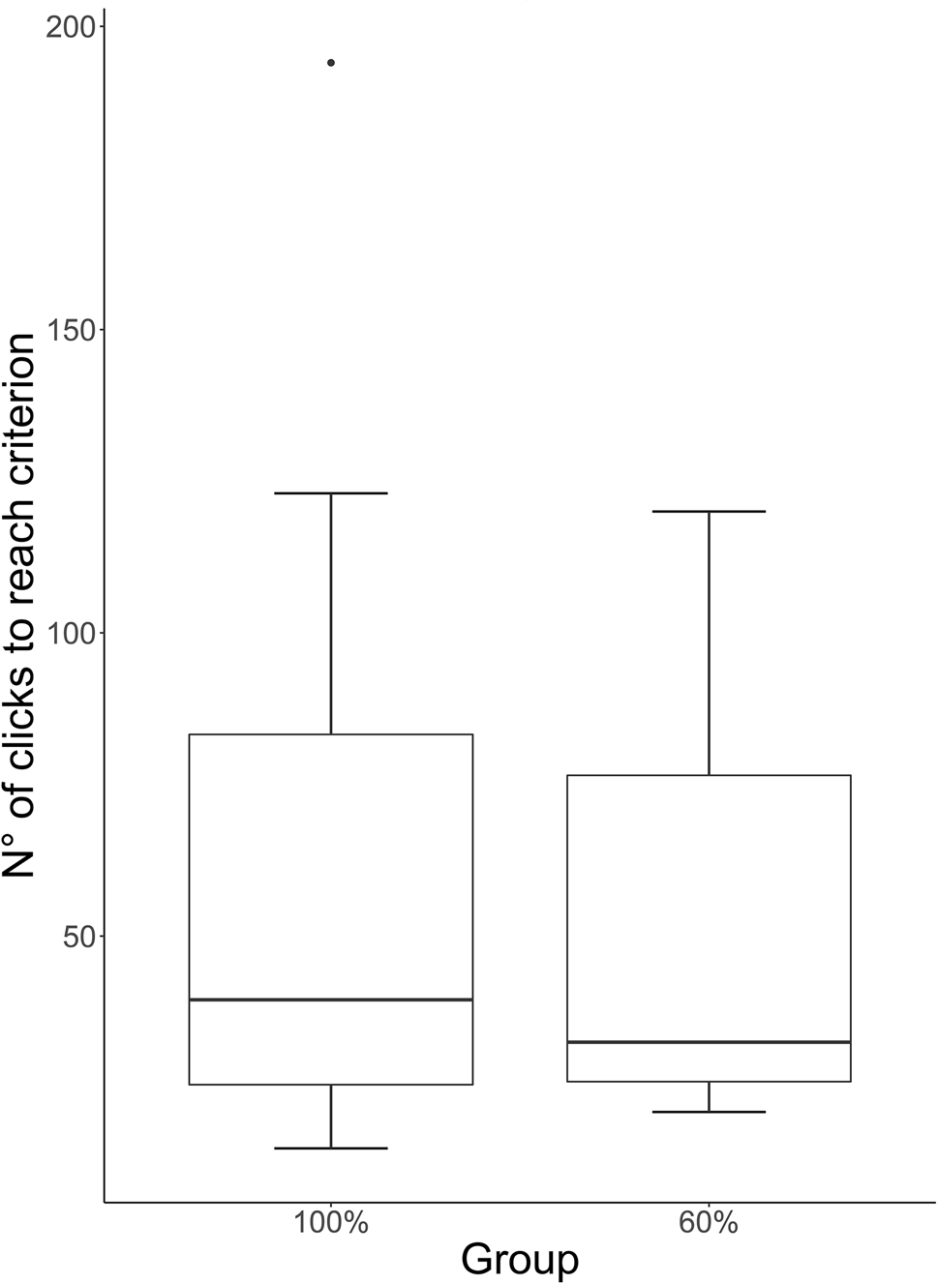
“Number of clicks to reach criterion” in the 100% and 60% rewarding groups: median and interquartile range (IQR; represented by the box), 25th percentile + 1.5 IQR, and 75th − 1.5 IQR (represented by the lower and the upper whiskers, respectively)

**Fig. 2:**
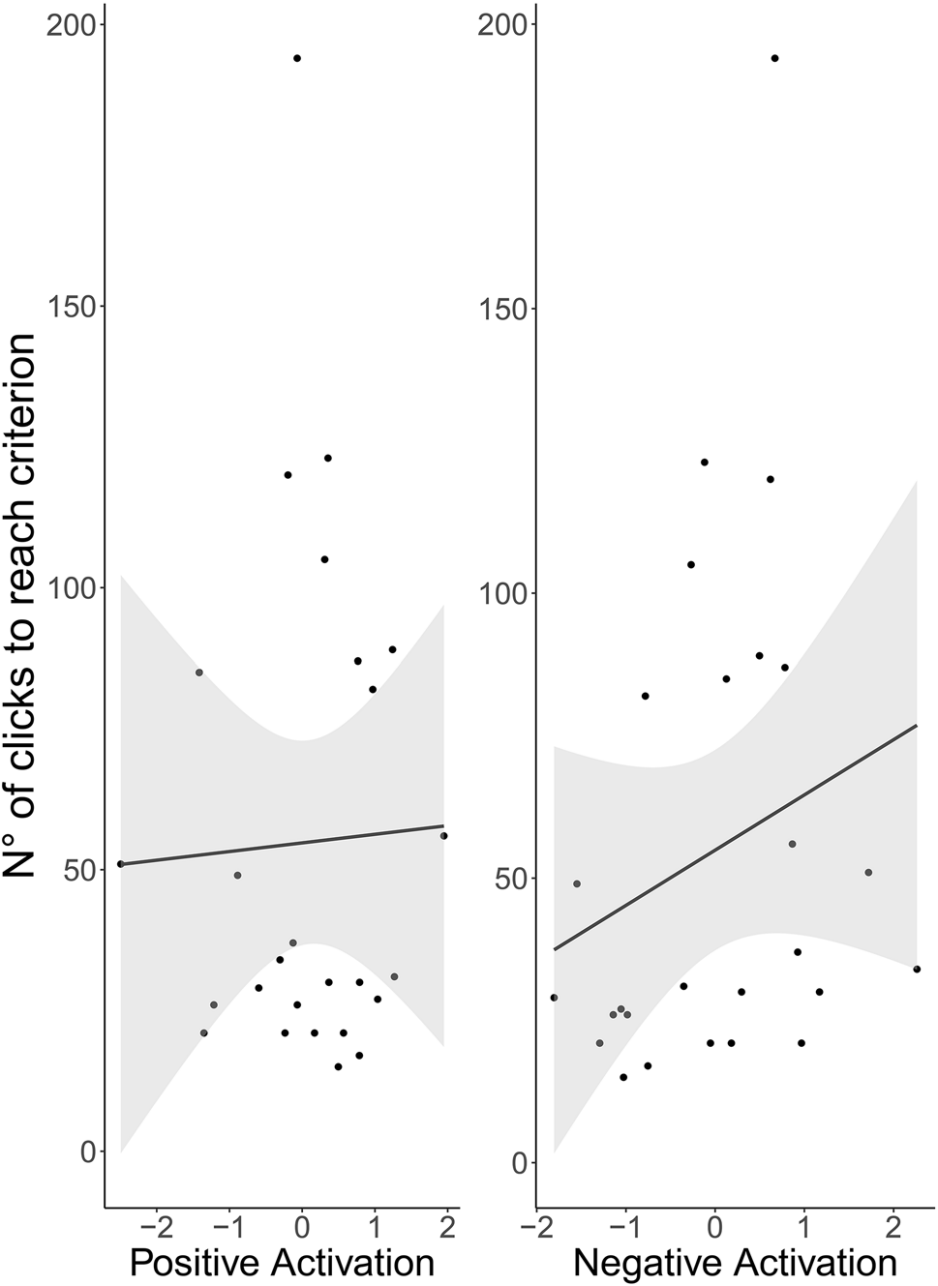
“Number of clicks to reach criterion” in relation to the two emotional reactivity components, shown for each dog (dots) plus regression line and 95% CI

Dogs needed a median of 32.50 trials to reach criterion (SD = 45.00, range 8–194 trials). Dogs of the 60% group were slightly faster than dogs of the 100% group (60%: median = 32.50, SD = 34.61, range = 21–120; 100%: median = 39.50, SD = 53.77, range = 8–194, Fig. 1) but the null-full model comparison did not reveal significance (*F* (5,17) = 0.34, *p* = 0.88), suggesting that none of the predictors influenced dogs’ learning speed (Table S2; Figs. 1, 2).

### Affective state

**Fig. 3:**
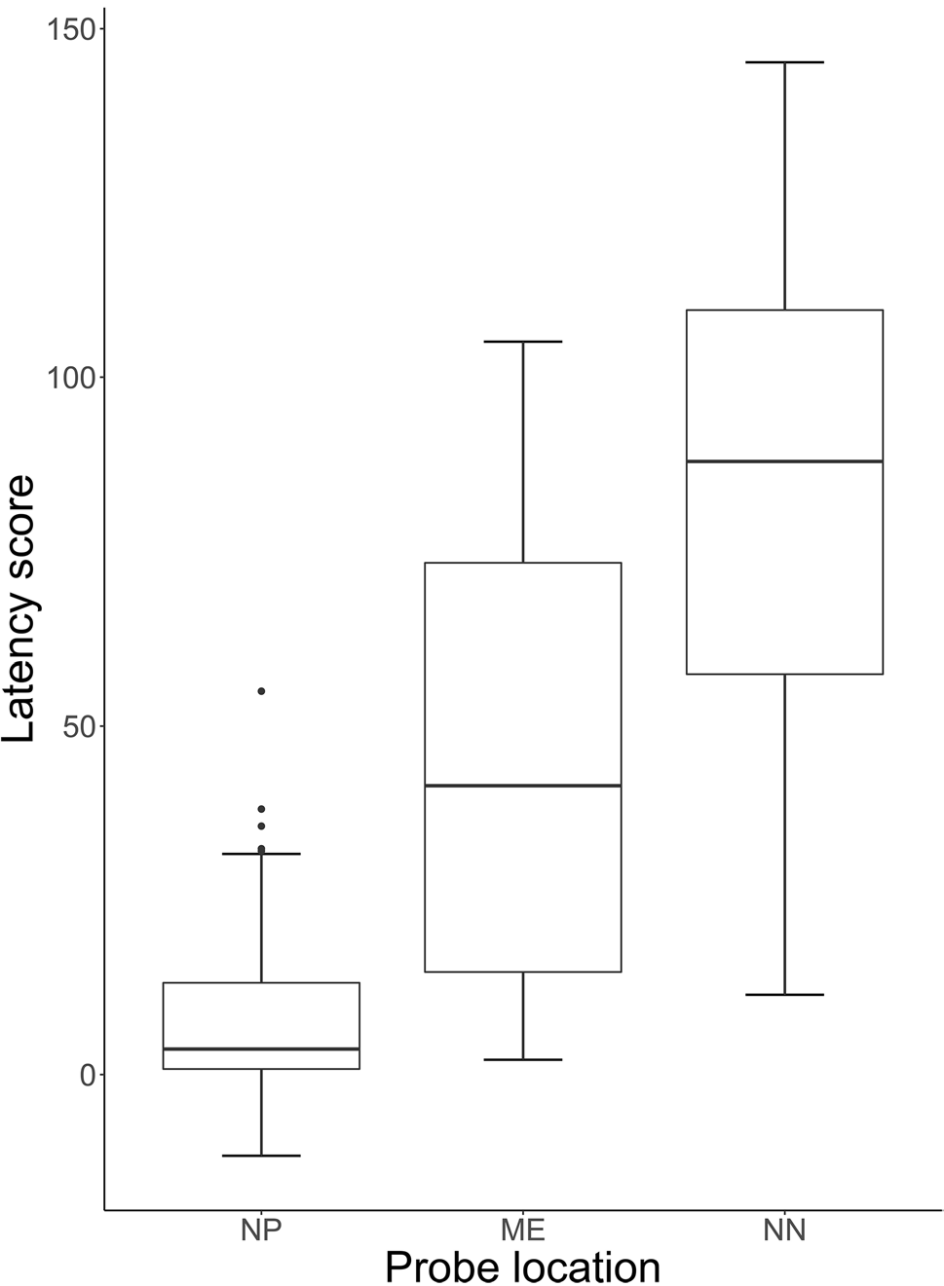
“Latency score” across the 3 probe locations: median and interquartile range (IQR; represented by the box), 25th percentile + 1.5 IQR, and 75th − 1.5 IQR (represented by the lower and the upper whiskers, respectively). *NP* near positive, *ME* middle, *NN* near negative

**Fig. 4:**
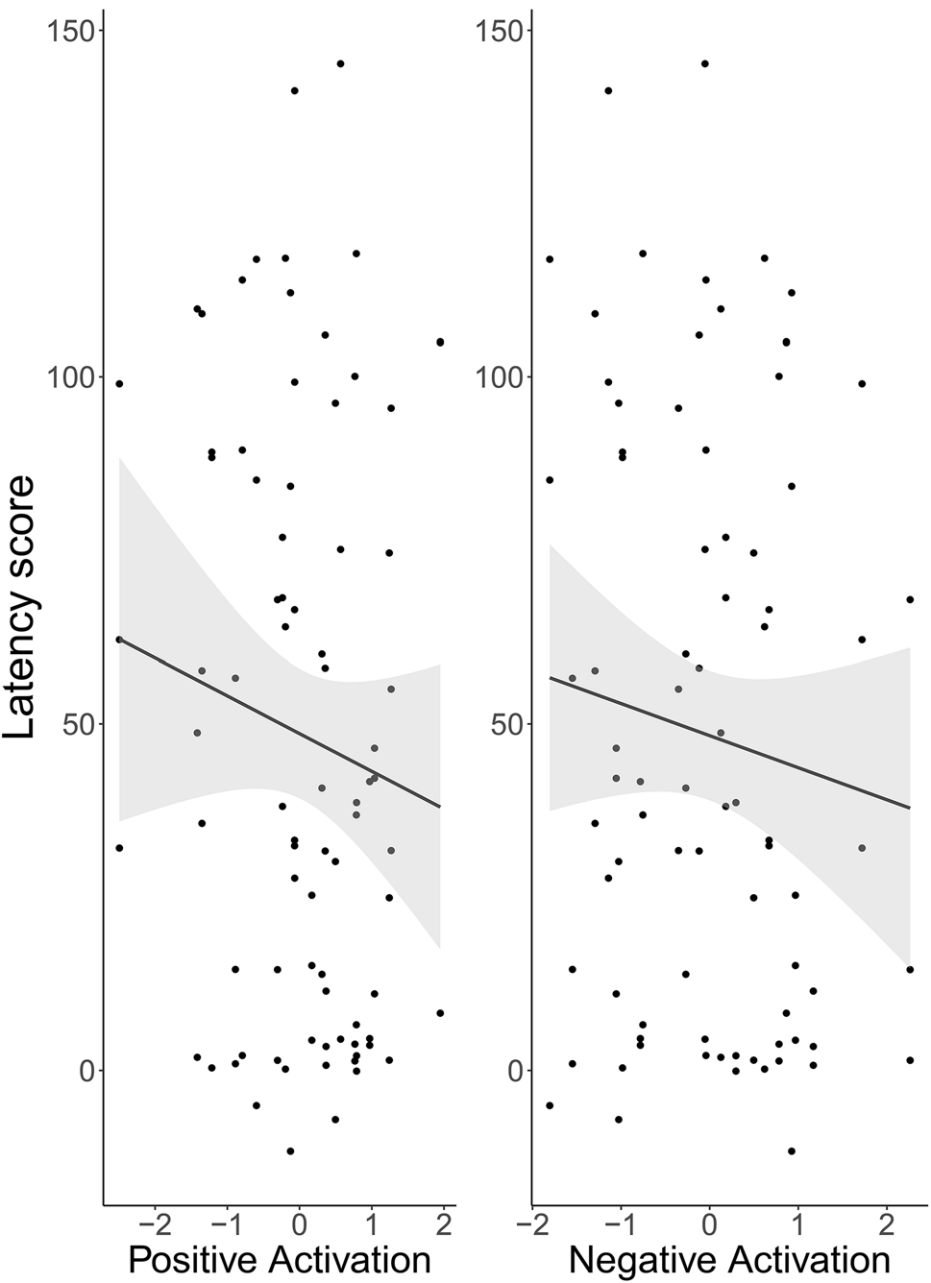
“Latency score” in relation to the two emotional reactivity components, shown for each dog (dots) plus regression line and 95% CI

The null-full model comparison using the latency score as response variable revealed a significant effect (*χ*^2^ = 80.71, *df* = 9, *p* < 0.001). None of the interactions were significant (all *p* > 0.05) and after removing them, we found a main effect of Location (*χ*^2^ = 70.29, *df* = 2, *p* < 0.001, Fig. 3), of Positive Activation (χ^2^ = 4.23, *df* = 1, *p* = 0.04, Fig. 4) and of Negative Activation (χ^2^ = 5.66, *df* = 1, *p* = 0.02, Fig. 4). In particular, dogs showed an increasing pessimistic bias (higher latency score) from probe NP to ME to NN (as expected from the test, estimates probe NN vs. probe ME: estimate ± SE = 34.75 ± 6.73, *t* = 5.16; probe NP vs. probe ME: estimate ± SE = − 37.26 ± 6.73, * t* = − 5.54) and a lower pessimistic bias at higher levels of emotional reactivity (Positive: estimate ± SE = − 7.25 ± 3.38, *t*  = − 2.14; Negative: estimate ± SE = − 7.68 ± 3.06, * t *  = − 2.52).

**Fig. 5:**
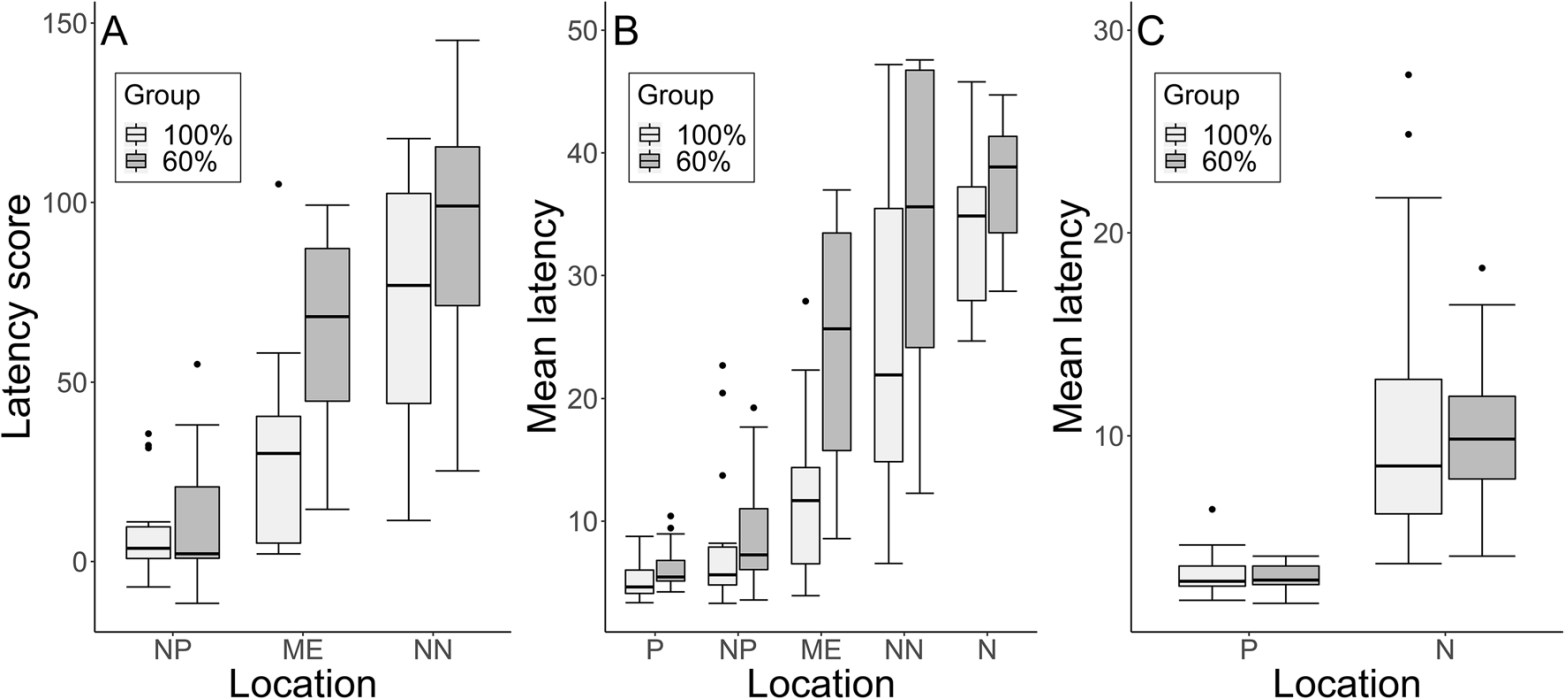
Results of the Cognitive Bias test: **a** “Latency score” across the ambiguous locations and 2 groups during the testing phase of the Cognitive Bias test; **b** “Mean latency” during the testing phase of the Cognitive Bias test (that is, after the last clicker training session) across all locations and 2 groups; **c** “Mean latency” during the refreshment phase (before the last clicker training session) of the Cognitive Bias test across the 2 training locations and 2 groups. For all: median and interquartile range (IQR; represented by the box), 25th percentile + 1.5 IQR, and 75th − 1.5 IQR (represented by the lower and the upper whiskers, respectively). *P* positive, *NP* near positive, *ME* middle, *NN* near negative, *N* negative

Although dogs of the 60% group had generally higher latency scores (NN (60%): median = 99.00, SD = 36.86; NN (100%): median = 76.85, SD = 32.63; ME (60%): median = 68.14, SD = 28.84; ME (100%): median = 30.14, SD = 28.65; NP (60%): median = 2.16, SD = 18.58; NP (100%): median = 3.69, SD = 13.25), we did not find a significant effect of Group (*χ*^2^ = 0.01, *df* = 1, *p* > 0.05, Fig. 5a; see Tables S3 and S4 for complete model results).

When considering the raw latency to reach the different locations during the testing phase of the Cognitive Bias test, we found a significant difference between the full and the null model (*χ*^2^ = 212.95, *df* = 13, *p* < 0.001). The interactions between the two questionnaire components and Group were not significant and were removed from the final model (both *p* > 0.05). The reduced model revealed a main effect of Positive Activation (χ^2^ = 10.91, *df* = 1, *p* = 0.001) and a significant effect of the interaction between Group and Location (χ^2^ = 10.14, *df* = 4, *p* = 0.04, Fig. 5b). In particular, dogs in the 60% group were slower in reaching the ME location than dogs in the 100% group (fitted value of the 60% not overlapping with the CI of the 100% group); see Tables S5, S6, and S7 for complete model results. Such differences between the two groups were not present during the refreshment phase (null-full model comparison: χ^2^ = 69.74, *df* = 5, *p* < 0.001). In fact, we did not find a significant effect of the interaction between Group and Location (*χ*^2^ = 59.74, *df* = 1, *p* > 0.05, Fig. 5c), nor a main effect of Group (*χ*^2^ = 57.79, *df* = 1, *p* > 0.05) on the dogs’ latency to reach the bowl. Instead, as expected, we found an effect of Location, with dogs being faster in reaching the P location than the N one (*χ*^2^ = 127.02, *df* = 1, *p* = 0.00); see Tables S8 and S9 for complete model results.

## Discussion

The goal of the present study was to evaluate whether dogs only partially rewarded during clicker training would learn faster and be negatively influenced in their affective state, than dogs that were continuously rewarded. In contrast to the hypothesis according to which a partial rewarding schedule increases motivation and attention thanks to the activation of the “seeking” system (Wise 2004; Berridge et al. 2009), here we found no difference between the two groups in terms of learning speed. Furthermore, our results also confirmed the hypothesis that different rewarding schedules would affect dogs’ performance in the cognitive bias test.

Our results regarding learning speed confirm previous evidence showing no difference in terms of behavior acquisition between animals continuously or partially rewarded (D’Amato et al. 1958; Fox and King 1961; Armus et al. 1962; Tombaugh 1970). In fact, in the present study, both groups of dogs learned the novel task with comparable speed. Overall, this suggests that continuous rewarding is not necessary to acquire a novel behavior when trained with a clicker, but also that training speed does not improve with the use of partial rewarding. Considering the high variability of responses (in terms of trials to reach criterion), with dogs needing between 8 and 194 trials to reach criterion, the lack of difference between the two groups cannot be explained by a ceiling (task too easy) or floor effect (task too difficult).

An explanation for the present results could be that a 60% schedule is not sufficient to have an effect of partial rewarding on learning (Burman et al. 2011). This schedule was chosen arbitrarily, as no similar study had been conducted using such procedures during clicker training. However, in a previous study, in which the secondary reinforcer was always provided, no difference was found between groups of rats rewarded 100% or 17% of the trials (Armus et al. 1962). Still, future studies are needed that parametrically vary the rewarding schedule to determine the functional relationship between the rewarding schedule and clicker training on a specific target behavior.

In the present study we used a shaping procedure (Ferster and Skinner 1957). That is, it was not always the same behavior being rewarded, rather, various behaviors, sequentially ordered in terms of approximations of the final behavior (e.g., from looking at the target to actually stepping on it). Such procedure is the one mostly used during clicker training (Pryor 1999) but it is also very different from what happens in laboratory studies (e.g., Berger et al. 1965; Egger and Miller 1962; Zimmerman 1957, 1971). In a context like the one of shaping, an individual is often not rewarded for a behavior that was previously successful, rather it is required to show something different. That is, a shaping procedure carries already quite a high level of uncertainty (it is intrinsically characterized by a constant partial reinforcement). This phenomenon could explain the difference in the results with the only previous study comparing partial and continuous rewarding in dogs, where dogs’ performance appeared hindered by the use of partial rewarding (Wennmacher 2007). In that study, in fact, dogs were asked to perform an already acquired behavior.

Despite the uncertainty inherent to the training process itself, we did find a difference between groups in cognitive bias performance. In fact, partially rewarded dogs were slower in reaching the ambiguous location than dogs continuously rewarded, suggesting that not receiving food after each click induces a pessimistic bias. Our findings are in line with a previous study reporting that dogs showed avoidant and stress-related behaviors when partially rewarded (Wennmacher 2007). Importantly, partially rewarded dogs’ lower speed in comparison to continuously rewarded ones was not due to an incidental unbalance between the two groups (e.g., a nonperfect match of breeds across groups). We could show this by having compared the latencies to reach the training locations during the refreshment phase of the Cognitive Bias test: directly before the onset of a clicker training session we found no difference in the dogs’ speed between the two groups.

Former studies have suggested that a difference in affective response between a continuous and a partial rewarding schedule appears only if rewarding is changed over time (switching from continuous to partial, Cuenya et al. 2012). This is because a first phase of continuous rewarding can create more expectations that would eventually be “betrayed” by a following switch to partial rewarding, than having been always exposed to a partial rewarding schedule (Cuenya et al. 2012; Burokas et al. 2012). Here we found that two independent groups of dogs, all naïve to the clicker, differed in terms of judgment of an ambiguous stimulus. Hence, even if dogs of the 60% group had never been exposed to other rewarding schedules in the context of clicker training beforehand, and had not built an expectation of always being rewarded after hearing the click, they were still negatively affected by the use of such a rewarding schedule. Future studies would need to investigate whether dogs first exposed to continuous rewarding and then switched to partial rewarding show slower learning and a more negative affective state than dogs that are always rewarded using the same schedule as in the present experiment.

One could wonder why we did not find a difference between the two groups when investigating the effect of treatment on the latency scores. A possible explanation for such apparently contradictory findings is that dogs of the 60% group were slower than dogs of the 100% group in reaching the negative location after the last clicker training session (although this difference was not significant). This might have affected the outcome of the mathematical calculation of the latency score (adjusted for the speed the dogs needed to reach the training locations), leveling the difference between the two groups.

A potential methodological aspect that might have made dogs in the 60% group slower than in the 100% group is that there could have been a larger temporal delay between the click and reward presentation in the partial rewarding group than in the continuous rewarding one. This might be because E needed to read whether the trial was a rewarded one or not in the 60% group (E had a list of trials reporting the rewarding schedule attached to her leg). We did so to minimize a potential bias arising from the fact that the experimenter was not blind to the treatment (i.e., E might have involuntarily avoided to click of a correct behavior knowing in advance that the trial was a nonrewarded one) by having the experimenter being unaware of whether the next click would have been followed by a reward in the partial rewarding group. Although we ensured that visual access to the paper sheet was optimized (the E was sitting on the floor with crossed legs and she only needed to look briefly downwards to consult the list), such a temporal delay might have led to increased frustration in dogs rewarded only 60% of the time. We suggest that future studies would take this element into account and standardize the temporal delay between the click and food delivery across groups.

It is important to notice that the Cognitive Bias test followed the treatment (last clicker training session) only after a break of 10 min and it lasted 45 min. Considering that this was the first study investigating the effect of different training procedures on dogs’ affective state, it is not possible to know whether the length of the break between treatment and testing might have dampened a potential treatment effect. Future studies varying such temporal delay between treatment and testing are needed to shed the light on this potential confounding factor.

One could argue that since having rewarded dogs from the partial rewarding group with larger rewards than dogs from the continuous rewarding group, it might have resulted in a higher excitement/reward anticipation that could have overridden any potential frustration effect arising from the partial rewarding schedule. However, the difference in weight between the food pieces used in the two groups was minimal (approximately 0.5g) and the effect we found was the opposite (dogs of the 60% group were slower than dogs of the 100% group) making such an explanation unlikely.

Moreover, we found a general effect of personality (specifically of dogs’ emotional reactivity) on affective state, with more reactive dogs (either to negative or positive novel events) showing a more optimistic bias than less reactive dogs. Associations between personality traits and cognitive bias have been already reported in various animal species (e.g., Asher et al. 2016; Cussen and Mench 2014), including dogs (Barnard et al. 2018). Moreover, humans not exposed to specific stimuli show a stronger effect of personality on such judgement bias than of mood state (Rusting 1999; Gomez et al. 2002). However, in the present study, higher scores on both emotional reactivity components (either to positive or negative stimuli) were associated with a less pessimistic bias. Importantly, the two components are not correlated with one another. One would expect that the effects would be opposite, with negative activation being associated with a more pessimistic bias (Rusting 1999) and positive activation with a more optimistic bias (Sharpe et al. 2011). Nevertheless, such results are in line with a previous study on dogs, showing that also some personality traits related to a negative activation (e.g., fear) were associated with a more optimistic bias (Barnard et al. 2018). Importantly, the two components should not be seen as opposite to one another (otherwise they would be negatively correlated), rather, they should be seen as expressions of different personality traits. In fact, dogs scoring higher in the negative activation component might be generally more sensitive to environmental stimuli. If so, having been exposed to a positive situation (that is, the clicker training sessions themselves) may have resulted in a larger improvement of their affective state, as compared to dogs scoring lower on negative activation. These results are supported by the fact that dogs’ latencies to reach the training locations during the refreshment phases (before the last clicker training session) were not affected by the emotional reactivity components. On the other hand, higher levels of positive activation seem to be mostly related to higher motivation and excitement that both, in turn, likely facilitate a more optimistic view of ambiguous stimuli. Both questionnaire components might reflect elevated levels of arousal/activity (independently from the valence of the stimulus eliciting the response) that might have been activated by the clicker session and have influenced the speed dogs needed to reach the ambiguous locations. In particular, dogs scoring higher on the emotional reactivity components could have become generally more active after training, therefore faster in reaching the bowl than dogs scoring lower.

To the best of our knowledge, this is the first study taking into account the potential role of personality in mediating the efficacy and consequences of different training techniques. For instance, instead of viewing differences in affective states as transitory, it would be important to investigate whether long-term affective biases (i.e., being more generally “optimistic” vs. “pessimistic”) have an influence on how an individual perceives and react to a specific treatment such as reward omission (Corr 2002; Gross et al. 1998). Combining these three different elements (i.e., individual differences, efficacy, and impact on welfare) is fundamental to design training methods that are tailored to the individual, not only to improve performance (e.g., for working dogs, Cobb et al. 2015; Feng et al. 2018), but also to improve the welfare of each animal.

To date, research on animal training has mostly focused on comparing the welfare implications of very different training methods (e.g., positive reinforcement-based vs. more coercive methods, see Ziv 2017 for a review) or comparing the efficacy of different techniques (using clicker vs. using only food, Chiandetti et al. 2016). To the best of our knowledge, no study has investigated whether technical differences within a training method that is generally considered as positive have an effect on dogs’ affective state. Despite some practitioners promoting the use of partial rewarding during clicker training, arguing that such technique increases the individual’s motivation and attention and improve training efficacy (McConnell 2014; Cecil 2016; but see Martin and Friedman 2011), the present results show that learning speed is not improved and that such methods could have a negative impact on dogs’ affective state. The present study has important welfare implications, providing evidence that dogs are sensitive to even subtle differences in training techniques and that caution should be exercised when designing training programs for both pet and working dogs.

## Electronic supplementary material

Below is the link to the electronic supplementary material.

Supplementary file1 (XLSX 23 kb)

Supplementary file2 (DOCX 32 kb)

## Data Availability

The full dataset is included as Supplementary Material.
