# Supplementary material for: Partial rewarding during clicker training does not improve naïve dogs’ learning speed and induces a pessimistic-like affective state
Source: Anim Cogn. 2020 Sep 8;24(1):107–19. doi: 10.1007/s10071-020-01425-9 (PMC7829239; doi:10.1007/s10071-020-01425-9)
Supplement: Supplementary file 2 — Supplementary file2 (DOCX 32 kb) [file 10071_2020_1425_MOESM2_ESM.docx]

**Authors:** Giulia Cimarelli^1,2*^, Julia Schösswender^1^, Roberta Vitiello^1,3,4,5^, Ludwig Huber^1^, Zsófia Virányi^1^

**Title:** Partial rewarding during clicker training does not improve naïve dogs’ learning speed and induces a pessimistic-like affective state

**Affiliations:**

^1^Clever Dog Lab, Comparative Cognition, Messerli Research Institute, University of Veterinary Medicine Vienna, Medical University of Vienna, University of Vienna, Austria

^2^Domestication Lab, Konrad Lorenz Institute of Ethology, University of Veterinary Medicine Vienna, Austria

^3^R(D)SVS, University of Edinburgh, Easter Bush, Midlothian, EH25 9RG, Scotland

^4^Scotland’s Rural University College (SRUC), Easter Bush, Midlothian, EH25 9RG, Scotland

^5^UMR PRC, INRA, CNRS, IFCE, Université de Tours, 37380 Nouzilly, France

**Corresponding author:** Giulia Cimarelli, Domestication Lab, Konrad Lorenz Institute of Ethology, University of Veterinary Medicine Vienna, Veterinaerplatz 1, 1210, Vienna, Austria. E-mail: [giulia.cimarelli@vetmeduni.ac.at](mailto:giulia.cimarelli@vetmeduni.ac.at). ORCID: 0000-0002-6031-0364

**Supplementary Material**

**List of questions of the emotional reactivity questionnaire (Sheppard & Mills 2002):**

Your dog…

- *Neg2: is rarely frightened

- Neg7: is easily startled by noises and / or movements

- Neg15: appears nervous and / or jumpy for several minutes after it has been startled

- Neg18: has a specific fear or phobia

- *Neg20: appears calm in noisy, crowded places

- Neg24: is frightened by noises from the television or radio

- *Neg25: usually appears relaxed

- *Neg27: adapts quickly to changes in its environment (e.g. being cared for by different people, moving house or a family member leaving home)

- Neg29: appears afraid of the vacuum cleaner or any other familiar household appliance

- *Neg39: appears calm in unfamiliar environments

- Neg40: appears unsettled by changes to its routine (e.g. if it is not fed at the usual time, if it is left alone for longer than usual)

- *Ene14: Your dog shows little interest in its surroundings

- Ene22: Your dog is full of energy

- *Ene30: Your dog is lazy

- *Ene34: Your dog requires a great deal of encouragement to take part in energetic activities

- Pers10: Your dog is very persistent in its efforts to get you to play

- Pers19: Your dog tries to escape from the garden

- Pers36: Your dog persists in being naughty despite being told off for the behavior

- Pers41: Your dog is very boisterous

- Exc1: Your dog becomes very excited when it is about to go for a walk (e.g. when it sees its lead, or when it hears “walkies”, etc.)

- Exc16: Your dog is easily excited

*These items scores were reversed before being included in the PCA.

**Results PCA**

| Table S1. Rotated Component Matrix | | |
| --- | --- | --- |
| Items | Component | |
|  | Negative Activation | Positive Activation |
| Neg27 | **0.710** | -0.044 |
| Neg40 | **0.681** | 0.445 |
| Neg2 | **0.667** | -0.160 |
| Neg7 | **0.664** | -0.055 |
| Neg24 | **0.628** | -0.124 |
| Neg15 | **0.628** | -0.357 |
| Neg18 | **0.599** | 0.081 |
| Neg39 | **0.591** | 0.322 |
| Neg29 | **0.576** | -0.228 |
| Neg25 | **0.551** | 0.550 |
| Neg20 | **0.532** | 0.023 |
| Ene22 | 0.180 | **0.775** |
| Pers10 | -0.034 | **0.715** |
| Ene30 | 0.107 | **0.678** |
| Exc16 | 0.138 | **0.527** |
| Pers41 | -0.249 | **0.524** |
| Ene14 | -0.193 | **0.503** |
| Exc1 | 0.118 | **0.394** |
| Pers36 | -0.144 | **0.340** |
| Pers19 | 0.057 | **-0.313** |
| Ene34 | -0.103 | **0.273** |
| Extraction Method: Principal Component Analysis. Rotation Method: Varimax with Kaiser Normalization. | | |

| Table S2. Learning speed: Results of the full model (estimates, standard errors, confidence intervals and estimates range obtained from one-by-one case exclusion) | | | | | | |
| --- | --- | --- | --- | --- | --- | --- |
| Predictor | Estimate | SE | 2.5% CI | 97.5% CI | Min | Max |
| Intercept | 3.92 | 0.28 | 3.34 | 4.51 | 3.81 | 4.06 |
| Group (60%) | - 0.22 | 0.38 | - 1.02 | 0.58 | - 0.38 | 0.13 |
| Positive Activation^1^ | - 0.18 | 0.28 | - 0.77 | 0.41 | - 0.39 | 0.02 |
| Negative Activation^1^ | 0.29 | 0.25 | - 0.23 | 0.81 | 0.12 | 0.52 |
| E Reactivation^1,2^ | 0.19 | 0.20 | - 0.22 | 0.60 | 0.05 | 0.31 |
| E Affiliation^1,2^ | 0.23 | 0.20 | - 0.19 | 0.65 | 0.03 | 0.36 |
| E Rejection^1,2^ | 0.06 | 0.15 | - 0.25 | 0.37 | - 0.08 | 0.12 |
| Group (60%) x Positive Activation^1^ | 0.22 | 0.33 | - 0.83 | 0.43 | 0.04 | 0.40 |
| Group (60%) x Negative Activation^1^ | - 0.20 | 0.30 | - 0.47 | 0.91 | - 0.41 | - 0.02 |
| ^1^variables centralized to a mean of zero and a standard deviation of 1. ^2^log-transformed variables. The original mean and standard deviation was the following: Positive Activation (m = 0.05, sd = 0.98), Negative Activation (m = - 0.01, sd = 1.02), E Reactivation (m = 26.93, sd = 41.54), E Affiliation (m = 14.17, sd = 10.41), E Rejection (m = 6.69, sd = 8.04). | | | | | | |

| Table S3. Affective state: results of the full model (estimates, standard errors, confidence intervals and estimates range obtained from one-by-one case exclusion) | | | | | | |
| --- | --- | --- | --- | --- | --- | --- |
| Predictor | Estimate | SE | 2.5% CI | 97.5% CI | Min | Max |
| Intercept | 38.32 | 9.22 | 20.96 | 56.11 | 32.25 | 43.12 |
| Group (60%) | 12.67 | 11.15 | - 8.98 | 34.75 | 8.44 | 22.04 |
| Probe NN^3^ | 41.98 | 9.49 | 24.54 | 61.31 | 37.04 | 45.81 |
| Probe NP^3^ | - 22.21 | 9.49 | - 39.65 | - 3.95 | - 24.16 | - 15.43 |
| Positive Activation^1^ | - 9.12 | 6.19 | - 21.99 | 2.80 | - 14.36 | 1.23 |
| Negative Activation^1^ | - 3.55 | 5.57 | - 14.64 | 6.92 | - 9.57 | 3.94 |
| E Reactivation^1,2^ | 17.14 | 4.21 | 8.15 | 25.01 | 13.99 | 20.10 |
| E Affiliation^1,2^ | - 10.64 | 4.36 | - 18.84 | - 1.69 | - 14.58 | - 7.74 |
| E Rejection ^1,2^ | 1.51 | 3.12 | - 5.04 | 7.76 | - 1.26 | 3.44 |
| Age^1^ | 0.08 | 3.34 | - 5.99 | 6.39 | - 2.78 | 3.21 |
| Sex (male) | 7.78 | 7.00 | - 5.56 | 22.17 | 0.91 | 14.60 |
| Group (60%) x Positive Activation | 1.87 | 7.25 | - 11.90 | 15.71 | - 7.58 | 6.22 |
| Group (60%) x Negative Activation | - 6.06 | 6.67 | - 18.98 | 8.38 | - 14.37 | - 0.57 |
| Group (60%) x Probe NN^3^ | - 13.43 | 12.93 | - 39.32 | 11.18 | - 17.26 | - 6.33 |
| Group (60%) x Probe NP^3^ | - 27.94 | 12.93 | - 51.85 | - 3.64 | - 34.73 | - 24.43 |
| ^1^variables centralized to a mean of zero and a standard deviation of 1. ^2^log-transformed variables. The original mean and standard deviation was the following: Positive Activation (m = 0.05, sd = 0.98), Negative Activation (m = - 0.01, sd = 1.02), E Reactivation (m = 26.93, sd = 41.54), E Affiliation (m = 14.17, sd = 10.41), E Rejection (m = 6.69, sd = 8.04), Age (m = 43.20, sd = 23.17). ^3^Dummy-coded with ME being the reference category. | | | | | | |

| Table S4. Affective state: Results of the reduced model, lacking the non-significant two-way interactions (estimates, standard errors, confidence intervals and likelihood ratio tests results) | | | | | | | |
| --- | --- | --- | --- | --- | --- | --- | --- |
| Predictor | Estimate | SE | 2.5% CI | 97.5% CI | X^2^ | df | p-value |
| Intercept | 42.80 | 7.22 | 27.74 | 57.00 |  |  |  |
| Group (60%) | 0.70 | 7.57 | - 14.34 | 16.25 | 0.01 | 1 | 0.93 |
| Probe NN^3^ | 34.75 | 6.73 | 21.12 | 47.61 | 70.29 | 2 | 0.00 |
| Probe NP^3^ | - 37.26 | 6.73 | - 50.13 | - 23.14 |  |  |  |
| Positive Activation^1^ | - 7.25 | 3.38 | - 13.74 | - 0.75 | 4.23 | 1 | 0.04 |
| Negative Activation^1^ | - 7.68 | 3.06 | - 13.65 | - 1.76 | 5.66 | 1 | 0.02 |
| E Reactivation^1,2^ | 17.00 | 3.97 | 9.05 | 24.48 |  |  |  |
| E Affiliation^1,2^ | - 10.00 | 4.15 | - 17.96 | - 2.13 |  |  |  |
| E Rejection ^1,2^ | 1.79 | 3.13 | - 4.70 | 7.94 |  |  |  |
| Age^1^ | 0.44 | 3.29 | - 5.56 | 6.82 |  |  |  |
| Sex (male) | 9.68 | 6.78 | - 3.83 | 22.48 |  |  |  |
| ^1^variables centralized to a mean of zero and a standard deviation of 1. ^2^log-transformed variables. The original mean and standard deviation was the following: Positive Activation (m = 0.05, sd = 0.98), Negative Activation (m = - 0.01, sd = 1.02), E Reactivation (m = 26.93, sd = 41.54), E Affiliation (m = 14.17, sd = 10.41), E Rejection (m = 6.69, sd = 8.04), Age (m = 43.20, sd = 23.17). ^3^Dummy-coded with ME being the reference category. | | | | | | | |

| Table S5. Mean latency to reach the bowl during the testing phase of the Cognitive Bias test: results of the full model (estimates, standard errors, confidence intervals and estimates range obtained from one-by-one case exclusion) | | | | | | |
| --- | --- | --- | --- | --- | --- | --- |
| Predictor | Estimate | SE | 2.5% CI | 97.5% CI | Min | Max |
| Intercept | 2.46 | 0.13 | 2.20 | 2.73 | 2.35 | 2.57 |
| Group (60%) | 0.39 | 0.16 | 0.07 | 0.71 | 0.30 | 0.57 |
| Probe N^3^ | 1.11 | 0.15 | 0.80 | 1.40 | 1.01 | 1.19 |
| Probe NN^3^ | 0.71 | 0.15 | 0.41 | 0.98 | 0.62 | 0.76 |
| Probe NP^3^ | -0.41 | 0.15 | -0.69 | -0.11 | -0.48 | -0.34 |
| Probe P^3^ | -0.78 | 0.15 | -1.08 | -0.50 | -0.84 | -0.71 |
| Positive Activation^1^ | -0.26 | 0.08 | -0.42 | -0.11 | -0.38 | -0.09 |
| Negative Activation^1^ | -0.11 | 0.07 | -0.25 | 0.02 | -0.20 | -0.02 |
| E Reactivation^1,2^ | 0.26 | 0.05 | 0.15 | 0.36 | 0.20 | 0.32 |
| E Affiliation^1,2^ | -0.06 | 0.06 | -0.17 | 0.05 | -0.16 | 0.00 |
| E Rejection ^1,2^ | 0.02 | 0.04 | -0.06 | 0.10 | -0.02 | 0.06 |
| Age^1^ | 0.02 | 0.04 | -0.06 | 0.11 | -0.00 | 0.09 |
| Sex (male) | 0.17 | 0.09 | -0.00 | 0.34 | 0.07 | 0.26 |
| Group (60%) x Positive Activation | 0.15 | 0.09 | -0.04 | 0.32 | -0.01 | 0.25 |
| Group (60%) x Negative Activation | 0.99 | 0.09 | -0.06 | 0.27 | -0.00 | 0.17 |
| Group (60%) x Probe N^3^ | -0.55 | 0.20 | -0.93 | -0.16 | -0.63 | -0.45 |
| Group (60%) x Probe NN^3^ | -0.33 | 0.20 | -0.69 | 0.05 | -0.39 | -0.22 |
| Group (60%) x Probe NP^3^ | -0.54 | 0.20 | -0.93 | -0.18 | -0.61 | -0.44 |
| Group (60%) x Probe P^3^ | -0.47 | 0.20 | -0.84 | -0.08 | -0.54 | -0.41 |
| ^1^variables centralized to a mean of zero and a standard deviation of 1. ^2^log-transformed variables. The original mean and standard deviation was the following: Positive Activation (m = 0.05, sd = 0.98), Negative Activation (m = - 0.01, sd = 1.02), E Reactivation (m = 26.93, sd = 41.54), E Affiliation (m = 14.17, sd = 10.41), E Rejection (m = 6.69, sd = 8.04), Age (m = 43.20, sd = 23.17). ^3^Dummy-coded with ME being the reference category. | | | | | | |

| Table S6. Mean latency to reach the bowl during the testing phase of the Cognitive Bias test: Results of the reduced model, lacking the non-significant two-way interactions (estimates, standard errors, confidence intervals and likelihood ratio tests results) | | | | | | | |
| --- | --- | --- | --- | --- | --- | --- | --- |
| Predictor | Estimate | SE | 2.5% CI | 97.5% CI | X^2^ | df | p-value |
| Intercept | 2.44 | 0.12 | 2.19 | 2.67 |  |  |  |
| Group (60%) | 0.44 | 0.16 | 0.14 | 0.77 |  |  |  |
| Probe N^3^ | 1.11 | 0.15 | 0.85 | 1.39 |  |  |  |
| Probe NN^3^ | 0.71 | 0.15 | 0.44 | 1.00 |  |  |  |
| Probe NP^3^ | -0.41 | 0.15 | -0.68 | -0.14 |  |  |  |
| Probe P^3^ | -0.78 | 0.15 | -1.06 | -0.49 |  |  |  |
| Positive Activation^1^ | -0.17 | 0.05 | -0.26 | -0.07 | 10.91 | 1 | 0.001 |
| Negative Activation^1^ | -0.08 | 0.04 | -0.16 | 0.01 | 3.13 | 1 | 0.08 |
| E Reactivation^1,2^ | 0.22 | 0.06 | 0.11 | 0.32 |  |  |  |
| E Affiliation^1,2^ | -0.04 | 0.06 | -0.14 | 0.07 |  |  |  |
| E Rejection ^1,2^ | 0.00 | 0.04 | -0.08 | 0.08 |  |  |  |
| Age^1^ | -0.00 | 0.05 | -0.08 | 0.08 |  |  |  |
| Sex (male) | 0.14 | 0.09 | -0.06 | 0.33 |  |  |  |
| Group (60%) x Probe N^3^ | -0.55 | 0.20 | -0.94 | -0.17 | 10.14 | 4 | 0.04 |
| Group (60%) x Probe NN^3^ | -0.33 | 0.20 | -0.74 | 0.03 |  |  |  |
| Group (60%) x Probe NP^3^ | -0.54 | 0.20 | -0.94 | -0.18 |  |  |  |
| Group (60%) x Probe P^3^ | -0.47 | 0.20 | -0.84 | -0.10 |  |  |  |
| ^1^variables centralized to a mean of zero and a standard deviation of 1. ^2^log-transformed variables. The original mean and standard deviation was the following: Positive Activation (m = 0.05, sd = 0.98), Negative Activation (m = - 0.01, sd = 1.02), E Reactivation (m = 26.93, sd = 41.54), E Affiliation (m = 14.17, sd = 10.41), E Rejection (m = 6.69, sd = 8.04), Age (m = 43.20, sd = 23.17). ^3^Dummy-coded with ME being the reference category. | | | | | | | |

| Table S7. Mean latency to reach the bowl during the testing phase of the Cognitive Bias test: results of the Group*Probe interaction emerging from the reduced model lacking the non-significant two-way interactions (fitted values and confidence intervals) | | | | | | | | | | |
| --- | --- | --- | --- | --- | --- | --- | --- | --- | --- | --- |
| Probe | Group | | Fitted values | | | 2.5% CI | | | 97.5% CI | |
| P | 100% | | 1.73 | | | 1.52 | | | 1.95 | |
|  | 60% | | 1.70 | | | 1.52 | | | 1.90 | |
| NP | 100% | | 2.10 | | | 1.86 | | | 2.32 | |
|  | 60% | | 2.00 | | | 1.81 | | | 2.21 | |
| ME | 100% | | 2.51 | | | 2.29 | | | 2.75 | |
|  | 60% | | 2.95 | | | 2.75 | | | 3.17 | |
| NN | 100% | | 3.22 | | | 3.00 | | | 3.43 | |
|  | 60% | | 3.33 | | | 3.12 | | | 3.53 | |
| N | 100% | | 3.63 | | | 3.40 | | | 3.87 | |
|  | 60% | | 3.52 | | | 3.32 | | | 3.73 | |
| Table S8. Mean latency to reach the bowl during the refreshment phase of the Cognitive Bias test: results of the full model (estimates, standard errors, confidence intervals and estimates range obtained from one-by-one case exclusion) | | | | | | | | | | |
| Predictor | | Estimate | | SE | 2.5% CI | | 97.5% CI | Min | | Max |
| Intercept | | 2.08 | | 0.13 | 1.86 | | 2.31 | 1.94 | | 2.17 |
| Group (60%) | | 0.06 | | 0.16 | -0.26 | | 0.35 | -0.05 | | 0.20 |
| Probe P^3^ | | -1.11 | | 0.14 | -1.38 | | -0.82 | -1.19 | | -1.04 |
| Positive Activation^1^ | | -0.05 | | 0.06 | -0.17 | | 0.07 | -0.08 | | 0.02 |
| Negative Activation^1^ | | -0.02 | | 0.05 | -0.12 | | 0.07 | -0.06 | | 0.01 |
| E Reactivation^1,2^ | | 0.05 | | 0.07 | -0.07 | | 0.17 | 0.00 | | 0.10 |
| E Affiliation^1,2^ | | 0.12 | | 0.07 | -0.03 | | 0.24 | 0.06 | | 0.17 |
| E Rejection ^1,2^ | | 0.01 | | 0.05 | -0.09 | | 0.12 | -0.05 | | 0.06 |
| Age^1^ | | -0.00 | | 0.06 | -0.11 | | 0.10 | -0.04 | | 0.03 |
| Sex (male) | | 0.19 | | 0.12 | -0.05 | | 0.41 | 0.08 | | 0.25 |
| Group (60%) x Probe P^3^ | | -0.05 | | 0.19 | -0.42 | | 0.34 | -0.13 | | 0.04 |
| ^1^variables centralized to a mean of zero and a standard deviation of 1. ^2^log-transformed variables. The original mean and standard deviation was the following: Positive Activation (m = 0.05, sd = 0.98), Negative Activation (m = - 0.01, sd = 1.02), E Reactivation (m = 26.93, sd = 41.54), E Affiliation (m = 14.17, sd = 10.41), E Rejection (m = 6.69, sd = 8.04), Age (m = 43.20, sd = 23.17). ^3^Dummy-coded with N being the reference category. | | | | | | | | | | |

| Table S9. Mean latency to reach the bowl during the refreshment phase of the Cognitive Bias test: Results of the reduced model, lacking the non-significant two-way interactions (estimates, standard errors, confidence intervals and likelihood ratio tests results) | | | | | | | |
| --- | --- | --- | --- | --- | --- | --- | --- |
| Predictor | Estimate | SE | 2.5% CI | 97.5% CI | X^2^ | df | p-value |
| Intercept | 2.10 | 0.11 | 1.87 | 2.32 |  |  |  |
| Group (60%) | 0.03 | 0.13 | -0.22 | 0.28 | 0.06 | 1 | 0.81 |
| Probe P^3^ | -1.14 | 0.10 | -1.33 | -0.97 | 69.28 | 1 | 0.00 |
| Positive Activation^1^ | -0.05 | 0.06 | -0.16 | 0.07 | 0.78 | 1 | 0.38 |
| Negative Activation^1^ | -0.02 | 0.05 | -0.13 | 0.08 | 0.22 | 1 | 0.64 |
| E Reactivation^1,2^ | 0.05 | 0.07 | -0.07 | 0.20 |  |  |  |
| E Affiliation^1,2^ | 0.12 | 0.07 | -0.02 | 0.25 |  |  |  |
| E Rejection ^1,2^ | 0.01 | 0.05 | -0.10 | 0.10 |  |  |  |
| Age^1^ | -0.00 | 0.06 | -0.12 | 0.11 |  |  |  |
| Sex (male) | 0.19 | 0.12 | -0.06 | 0.43 |  |  |  |
| ^1^variables centralized to a mean of zero and a standard deviation of 1. ^2^log-transformed variables. The original mean and standard deviation was the following: Positive Activation (m = 0.05, sd = 0.98), Negative Activation (m = - 0.01, sd = 1.02), E Reactivation (m = 26.93, sd = 41.54), E Affiliation (m = 14.17, sd = 10.41), E Rejection (m = 6.69, sd = 8.04), Age (m = 43.20, sd = 23.17). ^3^Dummy-coded with ME being the reference category. | | | | | | | |

**Models:**

*Learning speed*

*Model 1*

Null: log(clicks to reach criterion) ~ z.log.E Reactivation + z.log.E Affiliation + z.log.E Rejection

Full: log(clicks to reach criterion) ~ z.Negative Activation*group + z.Positive Activation*Group + z.log.E Reactivation + z.log.E Affiliation + z.log.E Rejection

*Affective state*

*Model 2 (testing phase)*

Null: Latency Score ~ z.age + sex + z.log.E Reactivation + z.log.E Affiliation + z.log.E Rejection + (1|subject)

Full: Latency Score ~ z.Negative Activation*Group + z.Positive Activation*Group + Location*Group + z.age + sex + z.log.E Reactivation + z.log.E Affiliation + z.log.E Rejection + (1|subject)

Reduced: Latency Score ~ z.Negative Activation + z.Positive Activation + Location + Group + z.age + sex + z.log.E Reactivation + z.log.E Affiliation + z.log.E Rejection + (1|subject)

*Model 3 (testing phase)*

Null: Mean Latency ~ z.age + sex + z.log.E Reactivation + z.log.E Affiliation + z.log.E Rejection + (1|subject)

Full: Mean Latency~ z.Negative Activation*Group + z.Positive Activation*Group + Location*Group + z.age + sex + z.log.E Reactivation + z.log.E Affiliation + z.log.E Rejection + (1|subject)

Reduced: Latency Score ~ z.Negative Activation + z.Positive Activation + Location*Group + z.age + sex + z.log.E Reactivation + z.log.E Affiliation + z.log.E Rejection + (1|subject)

*Model 4 (refreshment phase)*

Null: Mean Latency ~ z.age + sex + z.log.E Reactivation + z.log.E Affiliation + z.log.E Rejection + (1|subject)

Full: Mean Latency~ z.Negative Activation + z.Positive Activation + Location*Group + z.age + sex + z.log.E Reactivation + z.log.E Affiliation + z.log.E Rejection + (1|subject)

Reduced: Latency Score ~ z.Negative Activation + z.Positive Activation + Location + Group + z.age + sex + z.log.E Reactivation + z.log.E Affiliation + z.log.E Rejection + (1|subject)
